# Nicotinamide Adenine Dinucleotide Precursor Suppresses Hepatocellular Cancer Progression in Mice

**DOI:** 10.3390/nu15061447

**Published:** 2023-03-17

**Authors:** Nengzhi Pang, Qianrong Hu, Yujia Zhou, Ying Xiao, Wenli Li, Yijie Ding, Yunan Chen, Mingtong Ye, Lei Pei, Qiuyan Li, Yingying Gu, Yan Sun, Evandro Fei Fang, Mianrong Chen, Zhenfeng Zhang, Lili Yang

**Affiliations:** 1 Guangdong Provincial Key Laboratory of Food, Nutrition and Health, Department of Nutrition, School of Public Health, Sun Yat-sen University, Guangzhou 510080, China; 2 Department of Women Health Care, Guangzhou Baiyun District Maternal and Child Health Hospital, Guangzhou 510400, China; 3 Department of Immunization Programmes, Guangzhou Huadu District Center for Disease Control and Prevention, Guangzhou 510080, China; 4 Huizhou First Maternal and Child Health Care Hospital, Huizhou 516007, China; 5 Department of Clinical Molecular Biology, Akershus University Hospital, University of Oslo, 1478 Lørenskog, Norway; 6 Radiology Center, Translational Medicine Center, Guangzhou Key Laboratory for Research and Development of Nano-Biomedical Technology for Diagnosis and Therapy, Guangdong Provincial Education Department, Key Laboratory of Nano-Immunoregulation Tumor Microenvironment, Central Laboratory, The Second Affiliated Hospital, Guangzhou Medical University, Guangzhou 510260, China

**Keywords:** hepatocellular carcinoma (HCC), nicotinamide adenine dinucleotide (NAD), nicotinamide riboside (NR), cancer metastasis

## Abstract

Targeting Nicotinamide adenine dinucleotide (NAD) metabolism has emerged as a promising anti-cancer strategy; we aimed to explore the health benefits of boosting NAD levels with nicotinamide riboside (NR) on hepatocellular carcinoma (HCC). We established three in vivo tumor models, including subcutaneous transplantation tumor model in both Balb/c nude mice (xenograft), C57BL/6J mice (allograft), and hematogenous metastatic neoplasm in nude mice. NR (400 mg/kg bw) was supplied daily in gavage. In-situ tumor growth or noninvasive bioluminescence were measured to evaluate the effect of NR on the HCC process. HepG2 cells were treated with transforming growth factor-β (TGF-β) in the absence/presence of NR in vitro. We found that NR supplementation alleviated malignancy-induced weight loss and metastasis to lung in nude mice in both subcutaneous xenograft and hematogenous metastasis models. NR supplementation decreased metastasis to the bone and liver in the hematogenous metastasis model. NR supplementation also significantly decreased the size of allografted tumors and extended the survival time in C57BL/6J mice. In vitro experiments showed that NR intervention inhibited the migration and invasion of HepG2 cells triggered by TGF-β. In summary, our results supply evidence that boosting NAD levels by supplementing NR alleviates HCC progression and metastasis, which may serve as an effective treatment for the suppression of HCC progression.

## 1. Introduction

Liver cancer is the sixth most common cancer worldwide and was the third leading cause of cancer-related death globally in 2020 [1]. The most common type of liver cancer is hepatocellular carcinoma (HCC), accounting for ~90% of the cases [2]. Despite the availability of various treatment strategies for managing HCC, poor overall prognosis is often encountered because of the development of metastasis and recurrence [3]. Early-stage HCC tumors can be treated with curative options such as resection and local ablation, resulting in a 5-year survival rate of over 70%. However, symptomatic advanced-stage cases with portal invasion, distant metastasis, or lymph node metastasis have a median survival period of only 1–1.5 years [4,5]. Although hepatic resection can effectively treat early-stage HCC tumors, the recurrence of HCC remains a major concern due to the development of micro-metastases following resection and the emergence of de novo tumors in a highly carcinogenic microenvironment [6]. Post hepatic resection, a five-year recurrence rate has been reported to be as high as 70% [6]. Therefore, there is an urgent need to explore new strategies to repress the progression of HCC.

Nicotinamide adenine dinucleotide (NAD) is a coenzyme mediating redox reaction in various metabolic pathways. It also serves as a vital substrate for NAD-consuming enzymes such as sirtuins, PARPs, and CD38. NAD and its metabolites have been found to affect several biological processes, including those linked to cancer development, such as energy metabolism, DNA repair, epigenetic modifications, inflammation, stress resistance, and circadian rhythms [7]. Thus, NAD metabolism is a promising therapeutic target for cancer treatment [8,9]. Anti-cancer strategies targeting NAD metabolism aim to inhibit cancer cells’ survival and proliferation by reducing NAD levels, mainly through interfering with NAD-biosynthetic machinery [9,10]. Although some studies have reported that reduced NAD levels inhibit the migration and invasion ability of HCC cells in vitro [11,12], other studies have indicated that NAD depletion actually promotes tumorigenesis, tumor progression, and metastasis [13,14,15,16,17,18,19]. Moreover, boosting NAD with NAD precursors has been shown to suppress tumor development and metastasis both in vivo and in vitro [13,15,16,17]. To sum up, the role of NAD in the complex process of tumor development and metastasis is controversial, and it remains unknown whether NAD repletion can suppress or promote the development and metastasis of HCC.

Nicotinamide riboside (NR) is a potent NAD precursor that naturally exists in milk and has already been made available as a nutraceutical [20]. Oral NR has been shown to significantly elevate NAD levels in human blood and mouse liver [21]. NR supplementation has shown promising results in the therapy of numerous cardiovascular, neurodegenerative, and metabolic disorders [20]. Furthermore, previous studies have shown that NR supplementation can attenuate alcohol-induced liver injuries, protect against liver fibrosis, and promote liver regeneration [22,23,24]. However, only a few studies have investigated the potential prophylactic or therapeutic role of NR in cancer [13,25], and none have explored its effect on cancer metastasis.

In this study, we sought to test the effect of boosting NAD on tumor metastasis prevention. The findings suggest that NR supplementation could serve as a promising therapeutic strategy for preventing HCC progression.

## 2. Materials and Methods

### 2.1. Cell Culture and Treatment

Human liver cancer cell line HepG2 and murine hepatoma cell line Hepa1-6 were from American Type Culture Collection (ATCC). Human liver cell line LO2 was from the National Collection of Authenticated Cell Cultures. Cells were cultured in Dulbecco’s Modified Eagle Medium (DMEM) (Gibco^TM^, Thermo Fisher Scientific Inc., Waltham, MA, USA) supplemented with 10% fetal bovine serum (FBS) (Gibco^TM^, Thermo Fisher Scientific Inc., Waltham, MA, USA) and 1% penicillin-streptomycin (Gibco^TM^, Thermo Fisher Scientific Inc., Waltham, MA, USA) at 37 °C in a humidified, 5% CO_2_ atmosphere.

### 2.2. Animal Experiments

All animal experiments in this study were approved by the Animal Care and Protection Committee of Sun Yat-sen University (No. 2018-001 and 2022001319). The mice were purchased from Guangdong Medical Laboratory Animal Center (Guangzhou, Guangdong, China), housed in temperature-controlled animal facilities with 12 h of artificial light/dark cycles, and had ad libitum access to water and food. NR administrated in animal experiments was from ChromaDex (Irvine, CA, USA). The mice were sacrificed as long as the mouse had suffered from rapid weight loss of 15–20 percent within a few days or had shown a state of cachexia.

For the subcutaneous xenograft model, a total of 5 × 10^6^ HepG2 cells suspended in a 100 μL mixture of PBS and Matrigel (Corning, NY, USA) (1:1), were injected subcutaneously into the rear flanks of five-week-old male BALB/C nude mice. The tumor volumes were measured twice weekly using a digital vernier caliper and calculated as length × width^2^ × 0.52. When the tumor volume reached 300 mm^3^, mice were randomly subjected to the treatment of 400 mg/kg/day NR (NR group, *n* = 7) or saline vehicle (Control group, *n* = 6) by daily gavage. The body weights and tumor sizes of the mice were measured every two days.

For the hematogenous metastatic neoplasm model, GFP-and-luciferase-labeled HepG2 cells (2.5 × 10^6^ cells in 100 μL PBS) were injected into the lateral tail veins of five-week-old male BALB/C nude mice. Two hours after tumor cells injection, mice were injected i.p. with D-luciferin (150 mg/kg), then anesthetized and placed in an imaging chamber. Images (IVIS Spectrum, Perkin Elmer, Waltham, MA, USA) were taken to confirm whether an equal amount of tumor cells was successfully transfused. Mice were randomly subjected to the treatment of 400 mg/kg/day NR (NR group, *n* = 7) or saline vehicle (Control group, *n* = 7) by daily gavage the next day. To monitor the progression of tumor metastasis, mice were imaged by noninvasive bioluminescence every 16 days and before sacrifice. The lungs of mice were dissected and imaged. Quantification of bioluminescence was performed by Living Image 4.3.1 software (Perkin Elmer, Waltham, MA, USA).

For the subcutaneous allograft model, a total of 5 × 10^6^ Hepa1-6 cells were injected subcutaneously into the rear flanks of five-week-old male C57BL/6J mice. Seven days later, mice were randomly subjected to the treatment of 400 mg/kg/day NR (NR group, *n* = 9) or saline vehicle (Control group, *n* = 12) by daily gavage the next day. The tumor volumes were measured every three days.

All organ samples, including tumor samples, were collected and weighed, then immediately frozen and stored at −80 °C until analysis, or fixed in formalin for Hematoxylin-eosin (H&E) staining.

### 2.3. Human Samples

Human liver tissues were collected from patients pathologically diagnosed with HCC undergoing hepatectomy at affiliated hospitals of Sun Yat-sen University. The study was approved by the Human Research Ethics Committee of Sun Yat-sen University. Written informed consents were signed by patients before recruitment. Tissue samples were collected as HCC tissues and normal liver tissues (tissues far from the HCC tumor). All tissue samples were quickly frozen on dry ice or in liquid nitrogen and stored at −80 °C until analysis.

A total of 38 patients were recruited in this study. The demographic and pathological characteristics of patients are shown in Table S1.

### 2.4. Establishment of GFP- and Luciferase-Labeled HepG2 Cell Line

To establish the GFP and luciferase-labeled HepG2 cell line (HepG2-GL), HepG2 cells were transduced by ultra-purified lentivirus particles containing a plasmid encoding luciferase and eGFP (EX-hLUC-Lv201, Genecopoeia, Rockville, MD, USA) following the manufacturer’s protocol. After 72 h of transduction, puromycin was added into cell culture media at a final concentration of 2 μg/mL to select stably transduced cells. Puromycin-resistant cells were plated onto 96-well culture plates at a density of approximately one cell per well. A monoclonal cell line with the highest fluorescence intensity of GFP in fluorescence microscope view was expanded for further experiment. In addition, a dual-luciferase assay was conducted to check the firefly luciferase expression of HepG2-GL cells using Luc-Pair™ Duo-Luciferase Assay Kit 2.0 (Genecopoeia) following the manufacturer’s protocol.

### 2.5. Cell Viability Analysis

Cell viability was determined by the CellTiter 96^®^ AQueous Non-Radioactive Cell Proliferation Assay (G1112, Promega, Madison, WI, USA) composed of a novel tetrazolium compound (MTS) (Promega) and an electron coupling reagent (PMS) (P9625, Sigma-Aldrich, St. Louis, MO, USA). Cells were seeded on 96-well plates and NR (ChromaDex) was administrated for 48 h. Cell viability was measured by a microplate reader and quantified at 490 nm absorbance.

### 2.6. Migration and Invasion Assay

For wound healing assay, HepG2 cells were seeded in 12-well plates. Cells at 90–100% confluency were pretreated with 1 µg/mL Mitomycin C (HY-13316, MedChemExpress, Monmouth Junction, NJ, USA) for 1 h to exclude the effect of cell proliferation on the results of migration assays. Then, the cells were gently scratched with a sterile 10 μL pipette tip to create a wound. Cells were cultured in the FBS-free medium containing 4 ng/mL TGF-β1(240-B, R&D systems, Minneapolis, MN, USA) and 1 mM NR. The images of the migrating cells were captured at 0 h and 48 h after scratching by a light microscope (Leica, Wetzlar, Germany). The area of the wound was calculated with ImageJ 1.51j8 software. Values for analysis were expressed as the percentage of wound healing area to the initial wound area.

Transwell migration and invasion assay were performed using an 8.0 µm pore polycarbonate membrane insert (3422, Corning Inc., Corning, NY, USA) according to the manufacturer’s protocol. For the invasion assay, the insert was pre-coated with matrigel (356234, Corning Inc., Corning, NY, USA). HepG2 cells (2 × 10^4^ for migration, 3 × 10^4^ for invasion) in 200 µL of FBS-free medium containing 4 ng/mL TGF-β1 and 1 mM NR were cultured in the upper chambers, whereas 700 µL of DMEM (10% FBS) containing 4 ng/mL TGF-β1 was added to the lower chambers. After being incubated at 37 °C for 24 h, cells were fixed with 4% paraformaldehyde and stained with crystal violet. Images of cells on the bottom side of the inserts were taken for five random views, and the number of migrated or invaded cells was counted by ImageJ software.

### 2.7. Metabolites Quantification

For metabolites quantification, quick-frozen human HCC tissues were thoroughly grounded by mortar-grinder and homogenized with 80% methanol/water (*v*/*v*) solution by tissue lyser. After centrifugation, the vacuum-dried samples were resuspended in acetonitrile/water (*v*/*v*) solution. Metabolites of cells were extracted with 80% methanol/water (*v*/*v*) solution and vacuum dried. The metabolites were resuspended in acetonitrile/water (*v*/*v*) solution for LC/MS analysis. The LC/MS assay of the NAD metabolome was described previously [26]. Data were collected using UPLC (Agilent 1290, Agilent Technologies, California, USA)-TOF (Agilent 6538) and analyzed using Agilent MassHunter Qualitative B.07.00 software.

NAD metabolites were separated and analyzed using a Hypercarb column (100 × 2.1 mm, 3 μm, ThermoFisher Scientific, Waltham, MA, USA) in UPLC-QTOF System (Infinity/6538, Agilent Technologies, California, USA), as previously described [27].

NAD levels of mouse tumor tissues were measured using NAD+/NADH Assay Kit with WST-8 (S0175, Beyotime, Shanghai, China) according to the manufacturer’s instructions. The amounts of total NAD/NADH or NADH were measured at 450 nm absorbance by a microplate reader. The amount of NAD is equal to the total NAD/NADH content minus the amount of NADH.

### 2.8. Statistical Analysis

Results are presented as individual data points or mean ± standard error of the mean (SEM). Statistical analysis was performed using Graphpad Prism 8.0 software. Comparisons between groups were analyzed by unpaired *t* test, Mann–Whitney test, Kruskal–Wallis test, Mixed-effects model, or two-way ANOVA. *p* < 0.05 was considered statistically significant.

## 3. Results

### 3.1. Reduced Nicotinamide Adenine Dinucleotide (NAD) Level Was Found in Human Hepatocellular Carcinoma (HCC) Tissues

**Figure 1:**
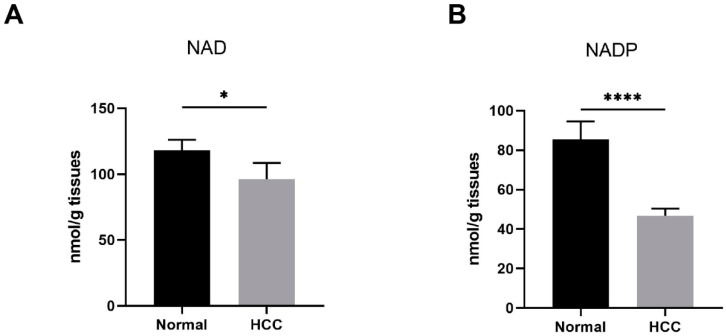
Reduced NAD levels were found in human HCC. (**A**,**B**) The NAD and NADP levels in human liver samples. Normal, liver tissues far from HCC nodules, *n* = 33. HCC, *n* = 38. * *p* < 0.05, **** *p* < 0.0001 compared to the normal tissues. *p*-values were calculated using Mann–Whitney test. HCC, hepatocellular carcinoma; NAD, nicotinamide adenine dinucleotide; NADP, nicotinamide adenine dinucleotide phosphate.

Cancer cells have a large demand for nicotinamide adenine dinucleotide (NAD) and its metabolites to support various crucial cellular processes, such as biosynthesis, energy metabolism, and antioxidant defense. Cellular NAD may be phosphorylated into nicotinamide adenine dinucleotide phosphate (NADP), whose reduced form, NADPH, provides the primary reducing power to eliminate ROS [7]. In this study, we examined NAD and NADP levels in human liver samples. NAD levels in HCC tissues were found to be significantly lower than those in normal liver tissues (Figure 1A). Similarly, NADP levels were remarkably decreased in HCC tissues compared to normal liver tissues (Figure 1B).

### 3.2. Nicotinamide Riboside (NR) Supplementation Alleviated Weight Loss Caused by Hepatocellular Carcinoma (HCC) in Immunodeficient Mice

**Figure 2:**
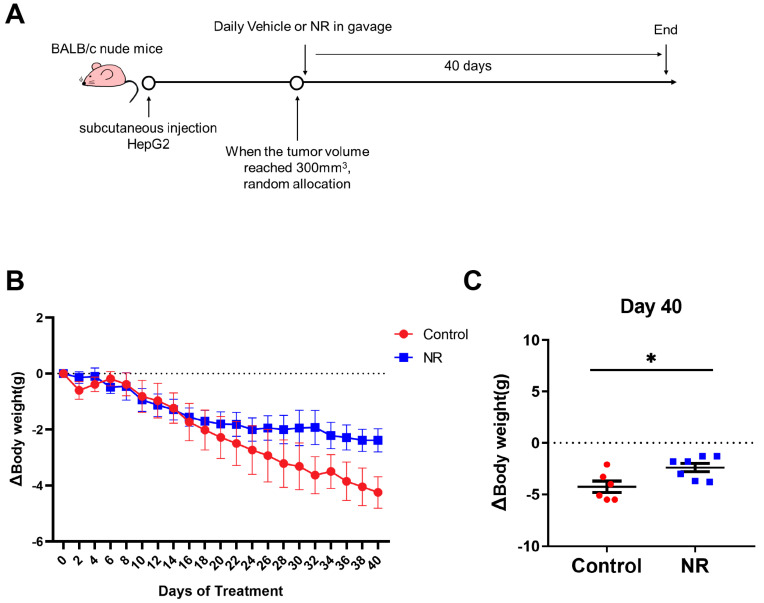
NR supplementation alleviated malignancy-induced weight loss in mice. (**A**) Schematic of the subcutaneous xenograft model. (**B**) Changes in weight loss of the mice during the intervention. (**C**) Differences in weight loss among two groups after 40 days of intervention. *n* = 6–7/group; * *p* < 0.05 compared to the control group. Dotted line represented the line (y = 0). Red dots: control mice; blue dots: NR-treated mice. *p*-values were calculated using two-way RM ANOVA test (**B**) and unpaired *t* test (**C**). NR, nicotinamide riboside.

Based on the reduction of NAD content in HCC tissues, we applied NR, a potent NAD precursor, to restore NAD levels for exploring the role of NAD in HCC progression. We established a subcutaneous cell-derived xenograft model and administrated NR via gavage (Figure 2A). At the beginning of the intervention, the body weights of mice in different groups were similar (Table 1). During the 40-day NR intervention, we found that NR-treated mice exhibited a significant reduction in weight loss compared to control mice (Figure 2B,C and Figure S1A). At the end of the intervention, the NR-treated mice had an 11.8% decrease in body weight, which was considerably lower than the 18.61% decrease from the control mice (Table 1). In addition, there was no difference in daily food intake among groups of mice during the intervention period, indicating that NR did not affect the appetite of the mice (Figure S1B). These results suggested that NR supplementation is able to alleviate the weight loss caused by HCC in this animal model.

### 3.3. Nicotinamide Riboside (NR) Supplementation Inhibited Spontaneous Lung Metastasis of Hepatocellular Carcinoma (HCC) without Inhibiting In Situ Tumor Growth in Immunodeficient Mice

**Figure 3:**
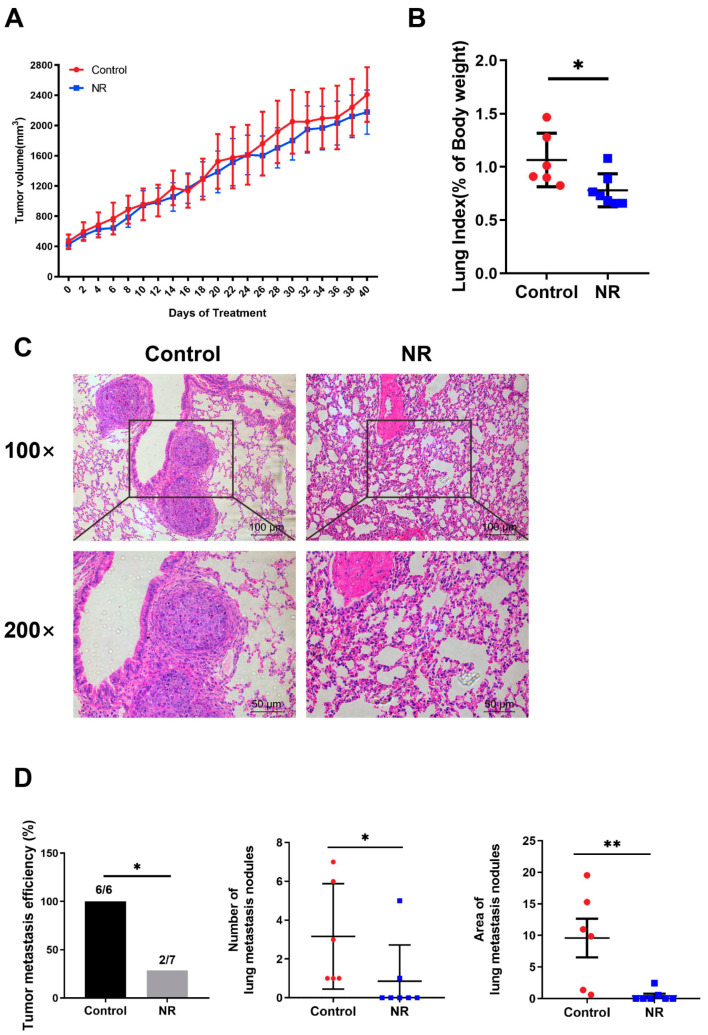
NR supplementation inhibited spontaneous lung metastasis of HCC tumors in mice without affecting in situ tumor growth. (**A**) Changes in subcutaneous tumor growth during the intervention. (**B**) Lung-to-body weight ratios at the endpoint of the intervention. (**C**) Lung metastasis nodules shown by representative H&E-stained sections. Scale bars: 100 μm (top row), 50 μm (bottom row). (**D**) The tumor metastatic efficiency, number, and area of lung metastasis nodules were quantified in H&E sections. *n* = 6–7/group; Red dots: control mice; blue dots: NR-treated mice. * *p* < 0.05, ** *p* < 0.01 compared to the control group. *p*-values were calculated using two-way RM ANOVA test (**A**), unpaired *t* test (**B**), Fisher’s exact test ((**D**)-tumor metastatic efficiency), and Mann–Whitney test ((**D**)-number and area). NR, nicotinamide riboside.

We investigated whether NR is able to relieve the tumor burden of mice by inhibiting in situ tumor growth. However, there were no statistical differences in both the tumor volume and the tumor index between the control and NR groups (Figure 3A and Figure S1C). NR increased the NAD levels of subcutaneous tumor of mice (Figure S1D). Cancer metastasis is an important part of HCC progression leading to the deterioration of physical health, including rapid weight loss. Due to this, we focused on whether NR supplementation suppressed tumor metastasis in the subcutaneous xenograft model. We found that the lung-to-body weight ratio was significantly higher in the control group compared to the NR group (Figure 3B). Considering the possibility of lung metastasis, we performed histological examinations and found that NR treatment significantly decreased the number and area of lung metastasis nodules in mice compared to the control mice (Figure 3C,D). The incidence rate of tumor metastasis was significantly lower in the NR (28.6%, 2/7) group comparing to the control group (100%, 6/6) (Figure 3D). These results suggested that NR supplementation is able to inhibit the spontaneous lung metastasis of HCC in the subcutaneous xenograft model.

### 3.4. Nicotinamide Riboside (NR) Supplementation Inhibited Hematogenous Multi-Organ Metastasis of Hepatocellular Carcinoma (HCC) in Immunodeficient Mice

**Figure 4:**
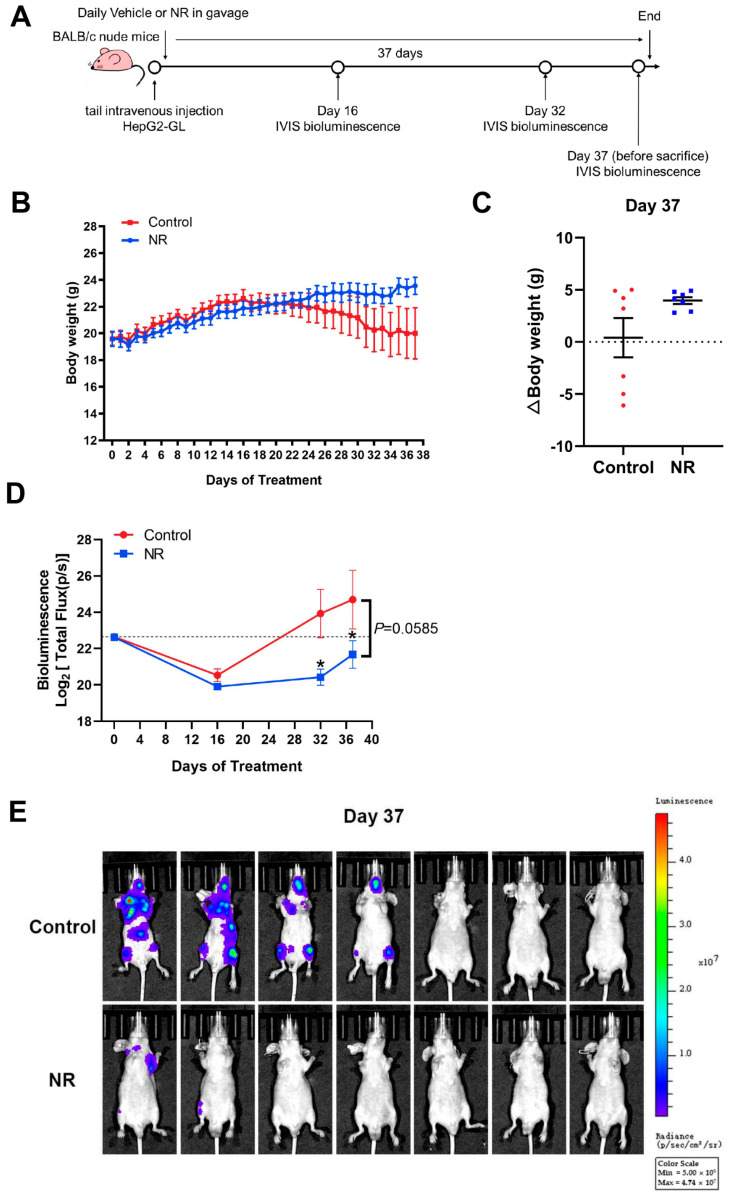

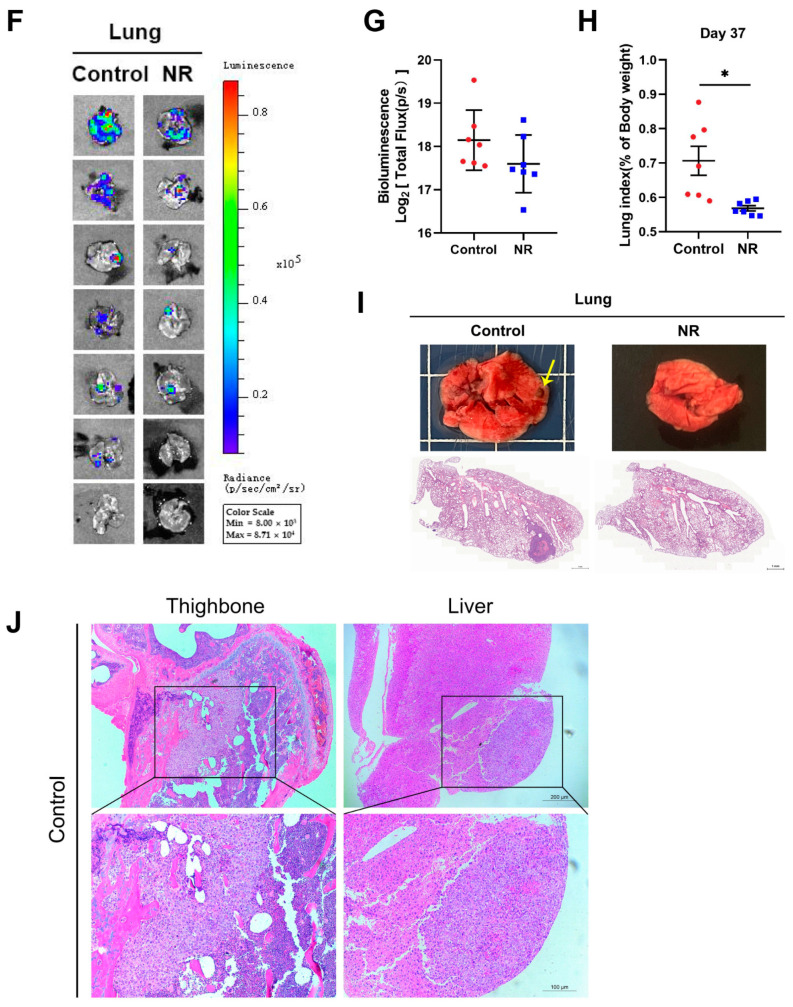
NR supplementation inhibited hematogenous multi-organ metastasis of HCC cells in mice. (**A**) Schematic of the hematogenous metastatic neoplasm model. (**B**) Trends in body weight of mice during the intervention. (**C**) Differences in weight changes between NR and control group after 37 days of intervention. Dotted line represented the line (y = 0). (**D**) The activity of HepG2-GL cells in vivo throughout the intervention, quantified by bioluminescent signals (Total Flux, p/s) of the whole mice body. Dotted line showed the bioluminescence signals on day 0. (**E**) The metastasis of HepG2-GL cells in vivo after 37 days of intervention shown by noninvasive bioluminescence imaging. (**F**) The metastasis of HepG2-GL cells in lungs after 37 days of intervention shown by bioluminescence imaging. (**G**) Quantification of the image in (**F**) with bioluminescent signals (Total Flux, p/s) of the lungs. (**H**) Lung-to-body weight ratios at the endpoint of the intervention. (**I**) Representative images of lungs dissected from mice per group. In the upper row, the arrow points to the macroscopic tumor nodule. In the lower row are scanning pictures of H&E staining sections, scale bars: 1 mm. (**J**) Representative H&E staining section of tumor metastasis to the bone and liver of mouse in the control group; scale bars: 200 μm (top row), 100 μm (bottom row). *n* = 7/group; Red dots: control mice; blue dots: NR-treated mice. * *p* < 0.05 compared to the control group. *p*-values were calculated using two-way RM ANOVA (**B**), unpaired *t* test and Welch’s correction (**C**,**H**), two-way RM ANOVA and Sidak’s multiple comparisons test (**D**), and Mann–Whitney test (**G**). NR, nicotinamide riboside.

To further validate the inhibitory effect of NR supplementation on cancer metastasis, we established a GFP and luciferase-labeled HepG2 cell line (HepG2-GL) and used the HepG2-GL cells to perform a hematogenous metastasis model. Bioluminescence imaging was applied to better monitor the metastasis process of HCC cells (Figure 4A).

Firstly, we found that NR supplementation contributed to the maintenance of body weight of tumor-bearing mice (Figure 4B). Overall, 42.9% (3/7) of mice in the control group suffered from dramatic weight loss in the late intervention period, whereas all of the NR-supplemented mice retained the ability to gain weight and remained active (Figure 4C). Next, we analyzed the noninvasive bioluminescence images throughout the experiment. At the begin of the experiment, mice in the NR and the control groups were successfully injected with HepG2-GL cells of the same viability and amount, confirmed by equal bioluminescence signals on day 0 (Figure 4D). After 32 days of intervention, the NR-treated mice were found to possess significantly lower overall tumor burden compared to the control mice, as shown by the bioluminescence signals (Figure 4D). It is worth noting that at the end of the intervention, 57% (4/7) of the control mice were found to suffer from distinctly more severe tumor burden compared to all of the NR-treated mice (Figure 4E). As for the bioluminescence data of lung, no notable difference could be found in quantified bioluminescence between the two groups (Figure 4F,G). Besides, the lung-to-body weight ratios were significantly higher in the control group compared to the NR group (Figure 4H). We found macroscopic pulmonary tumor nodule in the H&E staining of the control mice (Figure 4I). In addition, we found that the incidences of HCC cells metastasizing to the head, bone, and abdomen were all lower in the NR group compared to the control group (Figure 4J, Table 2), suggesting that NR was able to reduce the incidence of the multi-organ metastasis of HCC cells. Taken together, the results showed that NR is able to relieve tumor burden in mice by inhibiting multi-organ metastasis of HCC.

### 3.5. Nicotinamide Riboside (NR) Supplementation Suppressed the Subcutaneous Tumor Growth in Immunocompetent Mice

**Figure 5:**
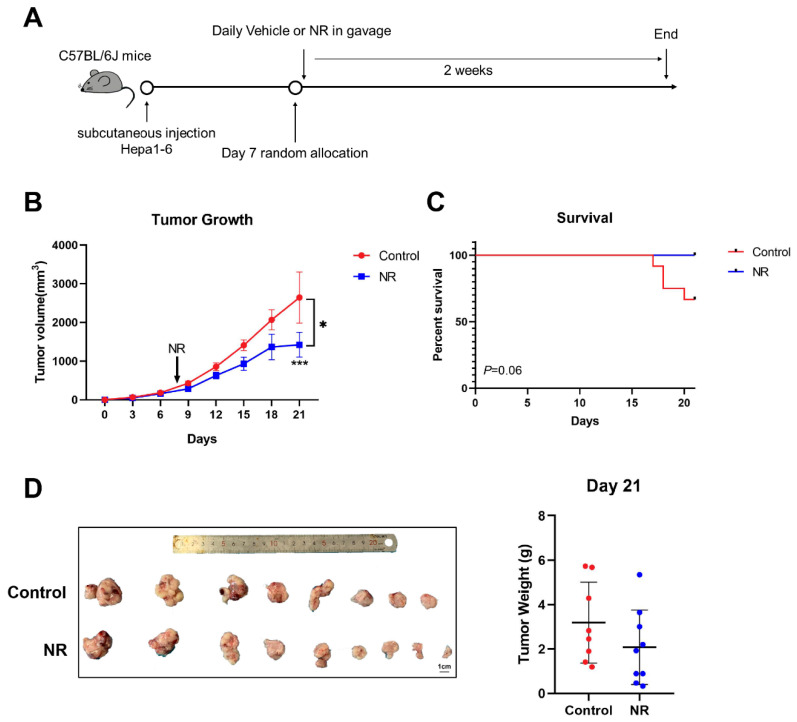
NR inhibited in situ tumor growth in C57BL/6J mice. (**A**) Schematic of the subcutaneous allograft model. (**B**) Subcutaneous tumor growth curves of mice during the experiment. (**C**) Survival percentage of mice in the experiment. (**D**) Tumor weight of mice after two weeks of treatment. *n* = 9–12/group; Red dots: control mice; blue dots: NR-treated mice. * *p* < 0.05 compared to the control group, *** *p* < 0.001 compared to the control group. *p*-values were calculated using mixed-effects model (REML) and Sidak’s multiple comparisons test (**B**), Log-rank (Mantel-Cox) test (**C**), and unpaired *t* test (**D**). NR, nicotinamide riboside.

Considering the potential anti-tumor effect of the immunity system in the tumor process, we also established subcutaneous cell-derived allograft models in immunocompetent mice and daily administrated NR supplementation in gavage (Figure 5A). During the two weeks of NR treatment, we found that NR significantly suppressed subcutaneous tumor growth in the NR-treated mice compared to the control mice (Figure 5B). NR supplementation prolonged the survival time of tumor-bearing mice (Figure 5C). Note that starting day 17, four of the mice in the control group were sacrificed due to rapid weight loss or obvious response lags. After two weeks of treatment, the numbers of smaller tumor were more in NR group than control group, however, the mean tumor volume was not statistically significant in two groups (Figure 5B,D).

### 3.6. Nicotinamide Riboside (NR) Inhibited the Migration and Invasion of HepG2 Cells

**Figure 6:**
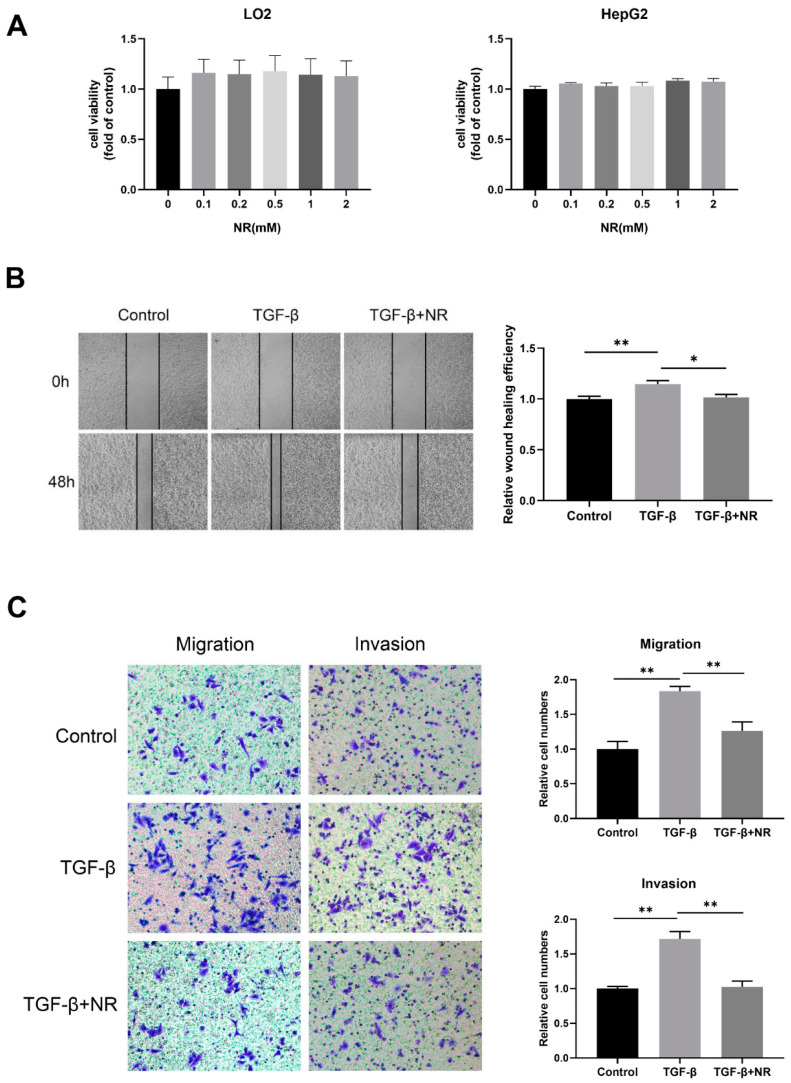
NR inhibited migration and invasion of HepG2 cells. (**A**) The cell viability of LO2 and HepG2 cells after 48-h NR treatment. (**B**) Wound-healing assay showing cell migration of HepG2 cells after 48 h. (**C**) Transwell assays showing cell migration and invasion of HepG2 cells after 24 h. *n* = 3; * *p* < 0.05, ** *p* < 0.01 compared to the TGF-β group. *p*-values were calculated using Kruskal–Wallis test and Dunn’s multiple comparisons test (**A**), one-way ANOVA and Tukey’s multiple comparisons test ((**B**,**C**)-migration), and one-way ANOVA and Tukey’s multiple comparisons test ((**C**)-invasion). NR, nicotinamide riboside; TGF-β, transforming growth factor-β.

We treated normal hepatocyte LO2 and HCC cell line HepG2 cells with different concentrations of NR and found that NR treatment at concentrations below 2 mM had no significant effect on the viability of LO2 and HepG2 cells (Figure 6A). These results suggested that short-term treatment of NR below 2 mM was not cytotoxic to either LO2 or HepG2 cells. For this reason, NR concentrations of 1mM were selected for subsequent experiments.

Transforming growth factor-β (TGF-β) is identified as one of the most potent inducers of epithelial–mesenchymal transition (EMT), which renders tumor cells more invasive and ultimately leads to tumor metastasis [28,29]. TGF-β decreased the NAD levels of HepG2 cells, however, the addition of NR to TGF-β-treated cells significantly restored the compromised NAD levels (Figure S3). To determine whether NR is able to inhibit the TGF-β-induced migration and invasion of HepG2 cells, we performed wound-healing and transwell assays. Our results revealed that the wound-healing ability of TGF-β-treated HepG2 cells was higher than that of control cells at 48 h, and this effect of TGF-β was reversed by NR supplement (Figure 6B). The transwell assay further confirmed that the migratory and invasion potential of HepG2 cells were notably elevated by TGF-β at 24 h, which were significantly reduced by NR treatment to the level of the control cells (Figure 6C). Taken together, this implies that NR is able to inhibit the migration and invasion of HCC cells triggered by TGF-β in vitro.

## 4. Discussion

In this study, we report for the first time that nicotinamide riboside (NR) supplementation could alleviate cancer metastasis in tumor-bearing mice and enhance the maintenance of body weight at the late stage of cancer. Our results reveal that NAD precursor may be a novel treatment for the prevention of HCC progression.

Our results showed that decreased levels of nicotinamide adenine dinucleotide (NAD) and nicotinamide adenine dinucleotide phosphate (NADP) were found in HCC tissues compared to non-carcinoma normal tissues. This suggests that NAD pool’s balance was disturbed during HCC formation. To explore the role of NAD in HCC progression, we established three different mice tumor models (with daily NR supply to replenish NAD pools). Surprisingly, we found that NR supplementation remarkably suppressed weight loss and cancer metastasis in immunodeficient mice. The energy metabolism of cancer cells is characterized by a shift of energy supply from mitochondrial respiration to glycolysis [30]. Note that glycolysis is a process where NAD production is low. During tumorigenesis, hyperproliferative tumor cells have acute demands of nucleic acid synthesis and NADPH [31]. This leads to the regeneration of NADPH, which consumes NADP [31]. NAD and NADP could possibly decrease during tumorigenesis. This corresponds to our finding of the obvious decrease in levels of NAD and NADP between HCC tissues and normal tissues.

NAD participates in the regulation of multiple cellular processes including energy metabolism, antioxidation, aging, and cell death [32]. The relationship between NAD and cancer is still elusive. Existing evidence indicates that the depletion of NAD through inhibiting the NAD salvage pathway can sensitize cancer cells to anti-tumor drugs, inhibit DNA repair, and trigger cell death [8]. Several studies reported that anti-tumor drugs in combination with inhibition of NAMPT, a rate-limiting enzyme in the NAD salvage pathway, can synergistically inhibit cancer cell proliferation [33]. NAD boosting as a strategy for treating cancer has caught some research spotlight recently. Prevention of the aging-related decline of NAD can hinder the metabolic reprogramming of tumors, which is the first step of tumorigenesis [34]. Moreover, literature research has demonstrated that inhibition of NAD de novo synthesis restrained PARP-1 activity, which subsequently suppressed DNA repair and led to liver tumorigenesis [13]. As mentioned, NR is a potent NAD precursor. In the mouse model, NR supplementation helped resume NAD pools and prevent DNA damage-induced liver tumorigenesis [13]. In breast cancer objects, supplementation of NAD precursors (niacin or nicotinamide) inhibited lung and multi-organ metastasis of tumors by inducing autophagy [15]. Consistent with these findings, our study observed that NR supplementation restored NAD pools and significantly inhibited cancer metastasis in both subcutaneous xenograft and hematogenous metastasis models. Our study also showed that NR supplementation suppressed in situ tumor growth in a subcutaneous allograft model established in immunocompetent mice. Taken together, our study indicated that NR supplementation can restore NAD pools, which in turn suppresses hepatocellular cancer progression in mice. This provides a new evidence to support the potential anti-tumor prospect via boosting NAD levels.

Cancer metastasis is the major cause of cancer-related death [35,36]. Patients with cancer metastasis are often accompanied with poor prognosis. The process of cancer metastasis is complex and dynamic. An increasing number of researchers have started to focus on the pro-metastatic function of transforming growth factor-β (TGF-β) during cancer progression [28,37]. TGF-β family proteins are able to induce epithelial-mesenchymal transition (EMT), a key mechanism of the initial cancer metastasis, via activating canonical SMAD and/or non-SMAD signaling pathway [37,38]. During EMT, tumor cells lose apical-basal polarity and cell–cell junctions and acquire front-back polarity; this causes the tumor cells to become more invasive [39]. Recently, a TGF-β-targeting drug has been identified as a promising therapy for cancer [40]. Previous studies confirmed that a decrease in NAD levels could affect the activities of NAD-dependent enzymes, such as the SIRT family proteins, and then promote the TGF-β signaling pathway [16,41]. Activating NAD-dependent enzymes or replenishing NAD by NAD precursors could attenuate TGF-β-related diseases [41,42,43,44]. In our study, we observed that TGF-β significantly promoted the migration and invasion of HepG2 cells in vitro, whereas NR supplementation was found to inhibit such effects. In future, we aim to further investigate the underlying mechanism of NR in counteracting TGF-β-triggered cancer metastasis.

We discovered that NR helped preserve the body weight of tumor-bearing mice. Ongoing body weight loss is a hallmark of cancer cachexia, which is defined as a multiorgan syndrome characterized by substantial weight loss [45,46,47,48]. The weight loss during cancer cachexia is primarily due to skeletal muscle and adipose tissues wasting on account of energy balance disorder and anorexia [45,46,47,48]. Cancer cachexia severely impairs life quality, weakens physical and emotional function, and reduces tolerability to anti-tumor therapy [49]. Patients with cancer cachexia also have poor clinical prognosis [49]. Improving nutrition status can help ameliorate cancer cachexia. For example, applying mirtazapine is a novel cancer adjuvant therapy. Mirtazapine could increase food intake and nutrition status in tumor-bearing mice, even though it had no significant effect on tumor growth [50]. In a model established by mouse colon carcinoma cells, preemptive intake of NR was found to significantly ameliorate cancer cachexia. Dietary intake of NR can prevent cancer-induced muscle atrophy and weight loss but not the growth of transplanted tumor [51]. Consistently, our study found no differences in tumor weight between the NR group and control in the subcutaneous xenograft model. Nevertheless, even though no food intake difference was found between the two groups, the loss of body weight in control was significantly higher than in the NR group. During intervention, we observed that the mice in the control group were depressed and exhibited response lags, whereas the mice in the NR group were more active in contrast. At the end of the experiment, mice in the control group had less subcutaneous fat content compared to the mice in the NR group (Figure S1E). This suggests that NR may be able to improve the nutrition status and life quality of advanced HCC mice.

In our xenograft model, no difference in transplanted tumor size was found between control and NR group. This could be due to: NR treatment did not start until the subcutaneous tumor volume exceeded 300 mm^3^; the high amount of HCC cells inoculated caused the speedy growth of the transplanted tumor; and once tumor has grown large enough (i.e., exceeded 300 mm^3^), anti-tumor medicine instead of nutrition supplement is required. Although we found significant differences in the number and area of lung metastatic nodules between the two groups via H&E-stained lung sections, this evaluation approach is rather limited. Bioluminescence imaging is more accurate in monitoring cancer metastasis. However, it is solely used in a mouse model established by fluorescent proteins or luciferase-labeled tumor cells. In our future studies, we aim to explore the protective effect of NR on transplanted tumor growth and cancer metastasis. This can be achieved by conducting a slow-growth xenograft model established by luciferase-labeled cells and a prophylactic NR intervention.

In our allograft model, transplanted tumor size was significantly lower in the NR group than in the control, which was different from the results in the xenograft model. This indicated that NR can suppress tumor growth in early tumorigenesis. Furthermore, this implied that anti-tumor immunity, especially T cells, might contribute to the inhibitory effect of NR on tumor growth. Taken together, these may explain why the inhibition of subcutaneous tumor growth by NR was limited to immunocompetent mice instead of nude mice lacking mature T cells in our study. However, in the allograft model, neither significant body weight loss nor lung metastasis was found in tumor-bearing mice (Figure S2). This could possibly be due to the fact that the experiment period was too short for advanced cancer to occur.

In summary, we found that replenishing NAD pools by NR could inhibit tumor growth and cancer metastasis as well as promote the overall health status of tumor-bearing mice. Our study revealed that boosting NAD by NR supplementation could be a novel strategy for the prevention of HCC progression. Our future studies will focus on the mechanisms of the effects of NR on HCC, and further clinical trials regarding NR are warranted.

## Figures and Tables

**Table 1:** The body weight of the mice and the percentage of weight loss during the intervention.

	Control	NR	*p*-Value *
Day 0 (g)	22.48 ± 0.73	20.46 ± 0.71	0.0729
Day 40 (g)	18.23 ± 0.24	18.07 ± 0.84	0.8584
% decrease	18.61%	11.80%	0.0412

* Data were analyzed by unpaired *t* test.

**Table 2:** Incidence of metastasis to different sites.

**Group**	**Head**	**Bone**	**Abdomen**
Control	4/7	4/7	2/7
NR	2/7	2/7	0/7

## Data Availability

The data presented in this study are available on request from the corresponding author.

## Supplementary Materials

The following supporting information can be downloaded at: https://www.mdpi.com/article/10.3390/nu15061447/s1, Supplementary figures: Figure S1: NR inhibited weight loss in tumor-bearing mice but not subcutaneous tumor growth. (A) Body weight curves of the mice during the intervention period. (B) Daily food intakes of the mice during the intervention period. (C) NR did not suppress tumor growth in immunodeficient mice. (D) NAD levels in tumor tissues of mice. (E) Respective photo of mice in two groups; Figure S2: Body weight curves of the mice during the intervention period; and Figure S3: NAD levels in HepG2 cells. * *p* < 0.05. Supplementary Table S1: The demographic and pathological characteristics of patients recruited in this study.
