# Supplementary material for: Nicotinamide Adenine Dinucleotide Precursor Suppresses Hepatocellular Cancer Progression in Mice
Source: Nutrients. 2023 Mar 17;15(6):1447. doi: 10.3390/nu15061447 (PMC10055624; doi:10.3390/nu15061447)
Supplement: Supplementary file 1 [file nutrients-15-01447-s001.zip › nutrients-2177401-supplementary.pdf]

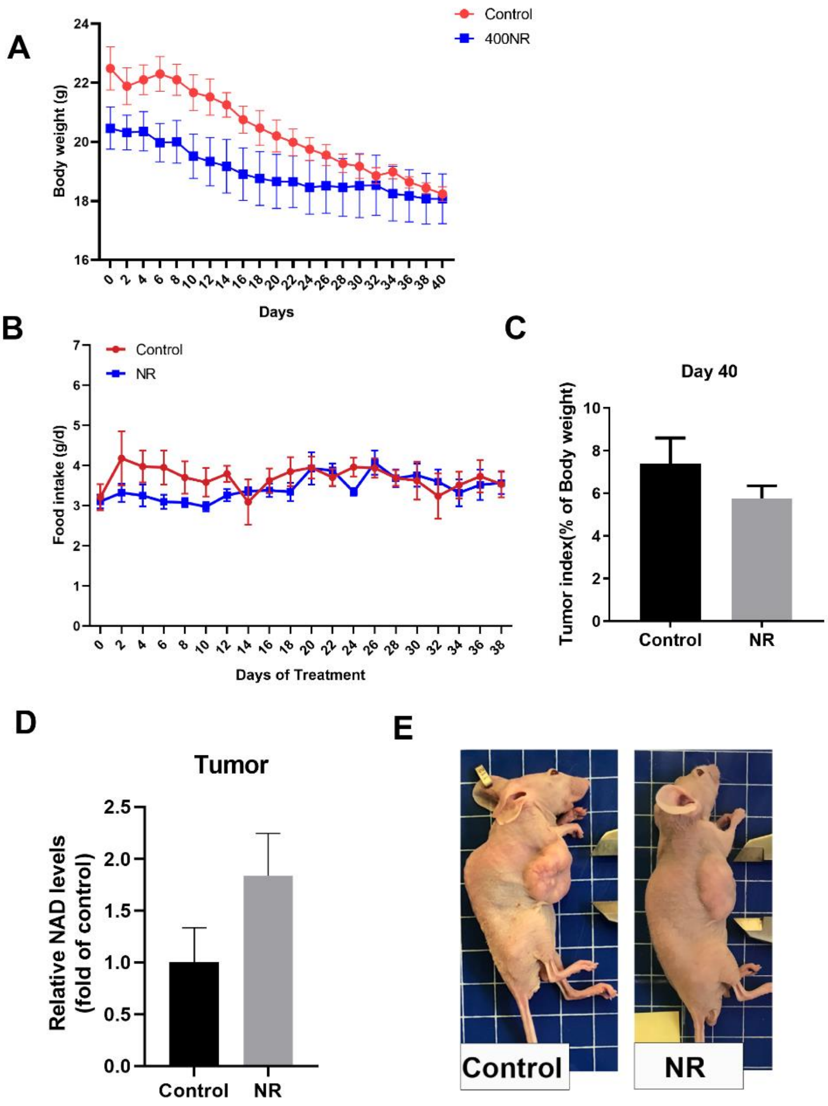

Figure S1. NR inhibited weight loss in tumor-bearing mice but not subcutaneous tumor growth.

(A). Body weight curves of the mice during the intervention period.

(B). Daily food intakes of the mice during the intervention period.

(C). NR did not suppress tumor growth in immunodeficient mice.

(D). NAD levels in tumor tissues of mice.

(E). Respective photo of mice in two groups.

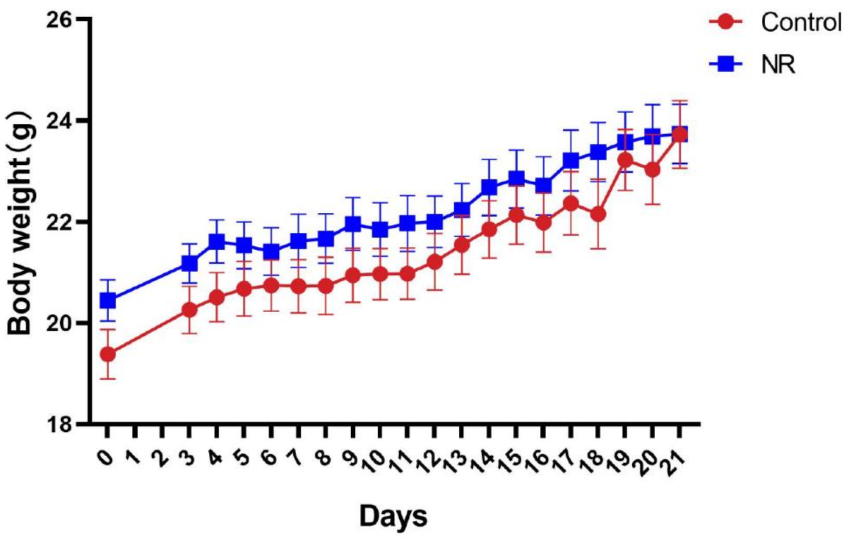

Figure S2. Body weight curves of the mice during the intervention period.

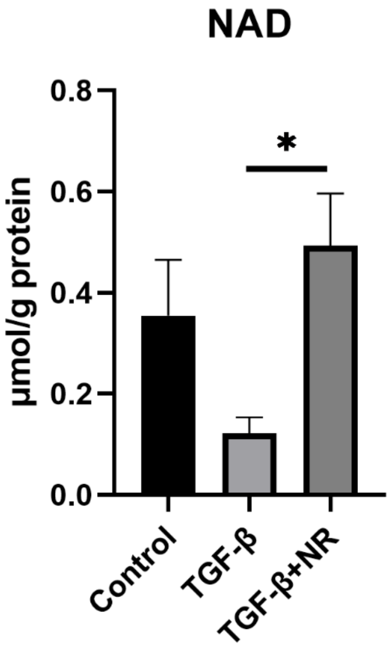

Figure S3. NAD levels in HepG2 cells. * p < 0.05

Table S1. The demographic and pathological characteristics of patients recruited in this study.

	The demographic and pathological characteristics of patients (n=38)
Gender	
Male	33
Female	5
Age	55.71±10.684
Virus infection	
Hepatitis B	17
Hepatitis C	1
None	20
AFP (ng/ml)	
<25.0	8
>25.0	6
Missing [a]	24

[a] Missing means missing of tracking data.
